# Optimal Experimental Design Based on Two-Dimensional Likelihood Profiles

**DOI:** 10.3389/fmolb.2022.800856

**Published:** 2022-02-23

**Authors:** Tim Litwin, Jens Timmer, Clemens Kreutz

**Affiliations:** ^1^ Institute of Medical Biometry and Statistics (IMBI), Faculty of Medicine and Medical Center, University of Freiburg, Freiburg, Germany; ^2^ Freiburg Center for Data Analysis and Modelling (FDM), University of Freiburg, Freiburg, Germany; ^3^ Institute of Physics, University of Freiburg, Freiburg, Germany; ^4^ Centre for Integrative Biological Signalling Studies (CIBSS), University of Freiburg, Freiburg, Germany

**Keywords:** experimental design, profile likelihood, systems biology, mathematical model, parameter uncertainty, prediction uncertainty, confidence distribution

## Abstract

Dynamic behavior of biological systems is commonly represented by non-linear models such as ordinary differential equations. A frequently encountered task in such systems is the estimation of model parameters based on measurement of biochemical compounds. Non-linear models require special techniques to estimate the uncertainty of the obtained model parameters and predictions, e.g. by exploiting the concept of the profile likelihood. Model parameters with significant uncertainty associated with their estimates hinder the interpretation of model results. Informing these model parameters by optimal experimental design minimizes the additional amount of data and therefore resources required in experiments. However, existing techniques of experimental design either require prior parameter distributions in Bayesian approaches or do not adequately deal with the non-linearity of the system in frequentist approaches. For identification of optimal experimental designs, we propose a two-dimensional profile likelihood approach, providing a design criterion which meaningfully represents the expected parameter uncertainty after measuring data for a specified experimental condition. The described approach is implemented into the open source toolbox Data2Dynamics in Matlab. The applicability of the method is demonstrated on an established systems biology model. For this demonstration, available data has been censored to simulate a setting in which parameters are not yet well determined. After determining the optimal experimental condition from the censored ones, a realistic evaluation was possible by re-introducing the censored data point corresponding to the optimal experimental condition. This provided a validation that our method is feasible in real-world applications. The approach applies to, but is not limited to, models in systems biology.

## 1 Introduction

With Fisher’s pioneering work on optimizing the design of agricultural experiments lying a century in the past, the design of informative experiments has long since become a foundation for most quantitative sciences. While there are undeniably practical aspects of conducting an experiment to generate the data used for analysis, planning a successful experiment requires consideration of statistical concepts even before any data is collected as this can help to develop the “logic of experimentation” (Bishop et al., 1982).

In systems biology, the underlying models used for analyses become increasingly complex. This is due to the fields aspiration to provide holistic descriptions of biological systems which are able to capture not only static properties of a system but the dynamic interactions of the system’s components (Kitano, 2002; Nurse and Hayles, 2011). For these systems, mathematical models are established to reduce the complexity of the biological components to their relevant features. The process of “building a model” is an intertwined process of finding a model which adequately describes the observed dynamics given the existing biological knowledge and providing the quantitative inputs for this model through experimentation (Kreutz and Timmer, 2009). The aim of systems biology is to construct “useful” models (Wieland et al., 2021), i.e. models that yield biological insights. Assessing whether a model is useful can be “notoriously difficult” (Liepe et al., 2013), even more so if the data obtained from experiments is insufficient to inform the model. Therefore, close cooperation of experimenters and theoreticians throughout the process increases the chance of generating data that is suitable for this task.

Biochemical processes can often be represented as ordinary differential equations (Nurse and Hayles, 2011; Liepe et al., 2013; Raue et al., 2013) which are often adequate representations of molecular dynamics. In general, this means that the observed biochemical compounds will be non-linearly related to the model parameters. Although such models are able to describe the system realistically, non-linearity proves to be a challenge in the analysis of the models properties. One consequence of non-linearity is the frequent absence of analytical solutions to the differential equations which determine the time-evolution of the biological states involved. Consequently, estimation of model parameters by optimization of the objective function, which measures the deviation of the model predictions to the measured data, is limited to numerical approaches (Raue et al., 2013). The difficulty of this “inverse problem” (Liepe et al., 2013) of determining the model parameters which describe the observed data the best is exacerbated in biological systems. Characterization of these systems can lead to a model with many parameters and biological states with the available data being noisy (Kreutz and Timmer, 2009). Additionally, the system is generally only partially observable, i.e. not all biochemical compounds in the model can be measured (Raue et al., 2009).

A major task in developing experiments in this defined setting is to propose practically feasible experiments which decrease the uncertainty about the value of parameters of interest. A well-known result from the classical theory of non-linear experimental design is that the optimal design depends on the “true model parameters,” i.e. the parameters that govern the true evolution of the system (Busetto et al., 2013), e.g. illustrated for the setting of Fisher’s dilution series experiments (Cochran, 1973). However, we are interested in inferring exactly these unknown parameters. A solid initial guess about the parameter values would solve the problem, but given the complex nature of the modeled systems, prior knowledge is usually sparse (Kreutz and Timmer, 2009; Bazil et al., 2012). A natural approach is then to design experiments sequentially (Cochran, 1973; Ford et al., 1989), i.e. measure the data in batches, updating the knowledge about the initial parameter values for each experimental design iteration.

Much of the classical literature on designing the optimal experiment is based on the Fisher information matrix (Ford et al., 1989; Atkinson and Donev, 1992; Fedorov, 2010). This is a natural approach in linear systems, as the inverted Fisher information matrix determines the covariance matrix of the estimated model parameters. Appropriate characteristics of this covariance matrix are then optimized by a suitable experimental design (Atkinson and Donev, 1992; Faller et al., 2003). However, application of the Fisher information matrix is known to be troublesome in non-linear systems if the amount of data is limited and statistical properties are far from asymptotic. The Wald confidence intervals implied by the Fisher information matrix might then only crudely reflect the existing uncertainty. Confidence intervals generated by the profile likelihood approach have more desirable properties in the finite sample case (Meeker and Escobar, 1995) and allow for the conceptional and operational definition of practical identifiability (Raue et al., 2009). Experimental planning in frequentist statistics should therefore make use of this powerful concept of quantifying parameter uncertainty and identifiability.

Approaches to the experimental design problem have also been developed in a Bayesian framework. The conceptual foundation of updating prior parameter knowledge given the newly measured data in Bayesian statistics provides natural solutions to the problem of experimental design. The information gain of an experiment can be reasonably quantified by means of the Shannon information (Lindley, 1956) and application of this theory to Bayesian experimental design provides a tool to plan optimal experiments for parameter inference (Huan and Marzouk, 2013; Liepe et al., 2013) and model discrimination (Busetto et al., 2013). However, we focus on a frequentist approach as it is usually not feasible to provide reasonable priors for all model parameter in the systems biology context.

There exist frequentist methods for experimental design if it is infeasible to provide prior information for all model parameters. If sets of parameters which are compatible with existing data about the system were known, the corresponding set of model trajectories would indicate for which observables and for which time points the model prediction is not yet reasonably constrained; such experimental conditions would then be “experimentally distinguishable” (Bazil et al., 2012). This set was previously constructed from efficient sampling of the parameter space (Bazil et al., 2012) or exploring the parameters along the likelihood profiles (Steiert et al., 2012). The latter method was applied in the DREAM6-Challenge (Dialogue for Reverse Engineering Assessments and Methods) and has been awarded as the best performing approach (Steiert et al., 2012). However, this approach assesses the impact of different sets of model parameters on the model predictions. In order to optimally design experiments which decrease parameter uncertainty, the logic of the design scheme has to be reversed: Instead of assessing the impact of different model parameters on the model prediction, the impact of different measurement outcomes on the parameter estimate of interest has to be assessed.

We propose a frequentist approach for optimal experimental design which realizes the full potential of the profile likelihood approach by extending the previously best-performing method (Steiert et al., 2012). For a specified experimental condition, we quantify the expected uncertainty of a targeted parameter of interest after a possible measurement. The parameter uncertainty after any specific measurement outcome is determined by the respective profile likelihood, effectively yielding a two-dimensional likelihood profile when accounting for different possible measurement outcomes. The range of reasonable measurement outcomes given the current data available before the measurement is quantified via the concept of validation profiles (Kreutz et al., 2012). The two-dimensional likelihood profile provides both the range of reasonable measurement outcomes of an intended experiment and their impact on the parameter likelihood profile. Hence, this allows for a definition of a design criterion which represents the expected average width of the confidence interval after measuring data for a certain experimental condition. The two-dimensional likelihood profiles therefore provide quantitative information usable for sequential experimental design and additionally serve as an intuitive tool to visualize the impact of an experiment on the uncertainty of the parameter of interest.

## 2 Materials and Methods

### 2.1 Mathematical Model

We introduce the concept of ordinary differential equation models, because they are frequently used for modeling of the dynamics of biological systems. However, we want to emphasize that the introduced method for experimental design is generic and only requires specification of a suitable likelihood function.

Biological quantities such as the concentration of a molecular compound are represented by mathematical states *x*(*t*) and are assumed to follow a set of ordinary differential equations

$$\dot{x}\left(t\right)=f\left(x,p,u\right)$$ (1)

which generates the trajectories according to the unknown underlying true dynamic model parameters *p*_0_. The function *f* is typically defined by translating biochemical interactions, e.g. by the rate equation approach. The trajectories depend on the specific experiment conducted which is denoted by the experimental perturbations *u*, representing interventions such as external stimulation of the system or knockout of specific genes. The set of model parameters will usually include the initial values *x*_0_ of the model states.

Estimation of the true parameters typically requires measurement of time-resolved data on these states. However, some states in the considered system might not be observable at all or only indirectly accessible, e.g. if only a sum of different states can be observed (Raue et al., 2009). Additionally, measured data will usually be subject to random errors. Therefore, the set of observables

$$y\left(t\right)=g\left(x\left(t\right),s_{obs}\right)+\epsilon$$ (2)

defines the types of data that can be measured. In this equation, *ϵ* describes the random error of the measurement which is usually assumed to be normally distributed, i.e. $N\left(0,\sigma^{2}\left(x\left(t\right),s_{err}\right)\right)$ , but not necessarily homogeneous across measurements, i.e. the magnitude of the noise might depend on parameters *s*_*err*_. Random variations in biological systems usually occur on a relative scale (Limpert et al., 2001) and are thus proportional to the current value of the state. This implies that errors are frequently normally distributed if the observables are considered on a logarithmic scale. The observation function *g* determines how the states are mapped unto the observables. This mapping will on many occasions introduce new unknown parameters *s*_*obs*_ such as scale parameters. The set of all parameters is denoted by *θ* = {*p*, *x*_0_, *s*_*obs*_, *s*_*err*_}.

The measured data of the system provides a set of scalar values *y*_*i*_ which each corresponds to an experimental condition *D*_*i*_ containing all information necessary to interpret the value *y*_*i*_. The experimental condition is uniquely defined as the measured observable, the time point of measurement and the corresponding experimental perturbation.

The objective function which indicates the agreement of experimental data with the model prediction given some parameters *θ* and measured data *Y* = {*y*_1_, …, *y*_*n*_} is the likelihood function

$$L\left(\theta |Y\right)=\underset{i}{\prod }\rho \left(y_{i}|D_{i},\theta \right)$$ (3)

with *ρ* indicating the probability density for the considered data point. Maximizing this likelihood leads to the maximum likelihood estimate $\hat{\theta }\left(Y\right)$ which indicates the parameters for which the fit between data and model predictions is optimal. Numerical optimization of this function is preferably performed by minimizing the monotonously transformed function *LL* = − 2 ln(*L*) to improve numerical stability. If the data is independently normally distributed and variances are known, this transformation has the advantageous properties that the optimization of the objective function is equivalent to least squares optimization.

### 2.2 Profile Likelihood

#### 2.2.1 Parameter Profile Likelihood

The task of parameter inference is not completed with the identification of the maximum likelihood estimate. In general, other parameter estimates may provide other model trajectories which might fit similarly well to the given data. Additionally, replications of the same experiment will lead to different measurement results and therefore also different parameter estimates due to variance in the biological samples and the measurement process. From a frequentist standpoint, methods are required to construct confidence intervals for either individual parameters or multiple parameters jointly, which have a pre-defined coverage probability of containing the true parameter value if the experiment were to be replicated. Within the context of this paper, we focus exclusively on confidence intervals for individual parameters.

The commonly encountered Wald confidence intervals are based on a quadratic approximation of the likelihood and fail if the model features non-linear dynamics (Meeker and Escobar, 1995; Raue et al., 2009). The quadratic approximation of the likelihood depends on the parametrization of the model, may not respect boundaries of the parameter space and cannot capture global behavior such as the existence of local optima.

A more refined tool which reduces the high-dimensional likelihood onto the one-dimensional parameter of interest *p*_*i*_ is the profile likelihood

$$PL_{p_{i}}\left(\beta |Y\right)≔-2\ln\left(\frac{L\left(\beta ,\hat{\omega }\left(\beta \right)|Y\right)}{L\left(\hat{\beta },\hat{\omega }|Y\right)}\right)$$ (4)

with the parameter vector *θ* = {*β*, *ω*} being split into the parameter of interest *p*_*i*_ = *β* and the nuisance parameters *p*_*i*≠*j*_ = *ω*. The hats indicate maximum likelihood estimates, i.e.

$$\hat{\omega }\left(\beta \right)=\underset{\omega }{\arg\max}\;L\left(\beta ,\omega |Y\right)$$ (5)

are the nuisance parameters which maximize the likelihood if *β* is fixed to a specific value. The parameter profile likelihood is invariant under one-to-one parameter transformations and can accurately reduce complex shapes of the underlying likelihood function to an adequate one-dimensional representation. Confidence intervals can be constructed from the parameter profile by Wilks’ Theorem (Wilks, 1938) and take the form

$$CI_{\alpha }=\left\{\beta |PL_{p_{i}}\left(\beta |Y\right)<icdf\left(\chi_{1}^{2},\alpha \right)\right\}.$$ (6)

with *icdf* representing the inverse cumulative distribution function. Note that high values of the profile likelihood defined in Eq. 4 correspond to lower values of the likelihood. This implies that parameter values *β* associated with a large profile likelihood value $PL_{p_{i}}\left(\beta |Y\right)$ are less likely to correspond to the true parameter value. Therefore, only parameter values with a profile likelihood value below a certain confidence threshold are included in the corresponding confidence interval.

Informally, Wilks’ theorem implies that asymptotically, these confidence intervals will attain the correct coverage probability *α* as they become equivalent to the Wald approximation. However, the finite sample properties of the profile likelihood intervals are superior. The notion of parameter profiles allows identifiability analyses on the parameters (Raue et al., 2009). Parameters can be: 1) Identifiable, in which case the width of the defined confidence interval is finite. 2) Structurally non-identifiable, in which case the profile likelihood is flat. This implies that any change of the parameter of interest can be compensated by changing other model parameters. 3) Practically non-identifiable, in which case the profile likelihood is not completely flat, but does not cross the confidence threshold to both sides such that the size of the confidence interval is infinite. While structural non-identifiability can only be resolved either by reparametrization of the model or qualitatively new experiments, practically non-identifiability can usually be resolved by providing higher quality data from similar experiments. Identifiability is distinct from the frequently encountered concept of sloppiness (Chis et al., 2016) which plays no role for the experimental design as discussed within this study. Due to the advantageous theoretical as well as practical properties of the profile likelihood, parameter uncertainties in this study are exclusively discussed in terms of their corresponding likelihood profile.

#### 2.2.2 Validation Profile Likelihood

The parameter profile likelihood allows for the evaluation of the uncertainty of parameters given the current data. For some applications, assessing the “prediction uncertainty,” i.e. the uncertainty about the outcome of measuring at a certain experimental condition, might be more relevant. In a frequentist setting, one can readily extend the concept of the parameter profile likelihood to this setting in the form of the “validation profile likelihood” (Kreutz et al., 2012), also called “predictive profile likelihood” (Bjornstad, 1990), in which case the likelihood is reduced to the dimension of the measurement outcome of interest. Formally, this profile is defined by

$$VPL\left(z|y\right)≔-2\ln\left(\frac{L\left(\hat{\theta }\left(Y,z\right)|Y,z\right)}{L\left(\hat{\theta }\left(Y\right)|Y,F\left(\hat{\theta }\left(Y\right)|D_{z}\right)\right)}\right)$$ (7)

with *z* defined as the outcome of measuring at experimental condition *D*_*z*_ and $F\left(\hat{\theta }\left(Y\right)|D_{z}\right)$ defined as the respective model prediction given parameters $\hat{\theta }\left(Y\right)$ . The interpretation of this validation profile likelihood is completely analogous to the parameter profile likelihood since the same coverage statement as in Eq. 6 holds (Kreutz et al., 2012) if the random variable *z* is normally distributed. It should be remarked that the statement about the coverage is slightly adapted in the sense that the coverage probability is true if *Y* and *z* are repeatedly drawn. Just as parameter uncertainty is reasonably quantified by the parameter profile likelihood, uncertainty of the measurement outcome for an experimental condition of interest is specified by the validation profile likelihood.

### 2.3 Experimental Design Task

Understanding the task of designing an informative experiment requires clarification. We start by introducing the common terminology of the theory of optimal experimental design. The design region K is the set of all experiments that can be conducted and a design point (or experimental condition) *D* within this design region labels a possible experiment which returns a data point (Fedorov, 2010). In our context, the design region K is designed as the set of all admissible triples of measurable observables *g*, possible time points of the experiment *t* as well as all external perturbations *u* considered. A design point *D* is then defined as the triple *D* = {*g*, *t*, *u*} (Kreutz et al., 2012). The collective of all conducted experiments can therefore be represented as the set of corresponding design points which is called the design of the experiment. This design is more commonly represented by a probability measure *ξ* on the design space K which compactly specifies all design points for which measurements outcomes are available. (Ford et al., 1989; Fedorov, 2010).

The problem we are concerned with is the reduction of uncertainty for a single parameter by conducting an experiment at an informative design point. This means that given a set of admissible design points, we want to decide which of these experimental designs will best reduce the existing uncertainty about a pre-specified parameter. To put this into a more formal framework, we are looking for a design criterion Φ:K→R which quantifies the most informative experiment in this context. Note that the optimal experimental design will usually depend on the unknown true parameters of the system. Additionally, we want to emphasize that we are concerned only with a single best experiment and not a batch of parallel experiments conducted at the same time.

### 2.4 Measuring Parameter Uncertainty

#### 2.4.1 Classical Theory

The optimal experimental design depends on the choice of a reasonable design criterion. Classical design theory solves this problem by applying the Fisher information matrix as the appropriate measure of information and establishing design criteria based on this matrix *M*(*θ*, *ξ*), i.e. the design criterion takes the form Φ(*M*(*θ*, *ξ*)) (Ford et al., 1989; Atkinson and Donev, 1992; Fedorov, 2010). The Fisher information matrix is concerned with the local behavior of the likelihood function around a specified parameter, which in application usually means in the neighborhood of the current maximum likelihood estimate of the parameters (Faller et al., 2003). For the same reasons discussed earlier, we propose that it is more adequate to utilize the profile likelihood of the parameter of interest *p*_*i*_ to construct a measure of information which we can use to design an optimal experiment.

#### 2.4.2 Confidence Distribution

There is no unique way to define the information available in the likelihood profile $\left\{\left(\beta ,PL_{p_{i}}\left(\beta \right)\right)|\beta \in \right\}$ of the parameter of interest *p*_*i*_. Instead of thinking in terms of the available information about the parameter, it is instructive to think in terms of existing uncertainty which we want to minimize by the experiment. In practical applications it is common that profile likelihoods are evaluated to obtain the respective 95%-confidence intervals *CI*_0.95_(*p*_*i*_) which serve as a measure of existing uncertainty. This comes with two notable issues: The 95%-interval might not be finite, which complicates the interpretation of existing uncertainty. This can be resolved in the definition of the model’s parameter space, which is constrained by parameter boundaries which span orders of magnitude and thus only weakly constrain the possible parameter values. On a more conceptual level, working with arbitrary fixed confidence levels is discouraged (Wasserstein and Lazar, 2016) and uncertainty is more comprehensively assessed if all confidence levels are considered simultaneously.

This issue can be resolved by confidence distributions (Xie and Singh, 2013) which can be thought of simultaneously containing the information about the confidence intervals to all levels. This concept allows the construction of an object that has the form of a distribution estimator for the parameter of interest *p*_*i*_ in the realm of frequentist statistics. The corresponding confidence density $\rho_{p_{i}}^{par}\left(\beta \right)$ has the property that each interval [*β*_1_, *β*_2_] which satisfies $\int_{\beta_{1}}^{\beta_{2}}\rho_{p_{i}}^{par}\left(\beta \right)d\beta =\alpha$ is an *α*%-confidence interval for the parameter *p*_*i*_. Conceptually, we can derive the confidence density $\rho_{p_{i}}^{par}\left(\beta \right)$ for a parameter from the set of confidence intervals {*CI*_*α*_(*p*_*i*_)|*α* ∈ [0, 1]} obtained from its likelihood profile. However, we will not use the concept explicitly and we remark that in the case of finite sample size, the obtained confidence distributions are not exact. By associating the parameter of interest *p*_*i*_ with their corresponding confidence distribution $\rho_{p_{i}}^{par}$ , we have a theoretically well-founded quantity on which the uncertainty of the parameter can be quantified.

#### 2.4.3 Uncertainty as a Scalar Quantity

Ranking different experiments by their information content requires a way to order their corresponding design criterion values. A necessary step to achieve this is to reduce the confidence distribution of a parameter to a scalar value. We suggest utilizing the average confidence interval width

$$\bar{w}\left(p_{i}|Y,z\right)=\int_{0}^{1}w\left(CI_{\alpha }\left(p_{i}|Y,z\right)\right)d\alpha$$ (8)

to summarize the information content of the confidence distribution. The function *w* assigns the width to the corresponding confidence interval. Different confidence interval widths are averaged by weighting with their respective confidence measure *dα*. The measure *dα* specifies the confidence that the true parameter value is covered by the interval *CI*_*α*+d*α*_(*p*_*i*_), but not by *CI*_*α*_(*p*_*i*_). Evaluation of this average confidence interval width does not require the explicit confidence distribution but only the individual confidence intervals. Thus, it can be directly calculated from the profile likelihood. In practice, we will only consider confidence intervals up to the 95%-level to ensure practical feasibility.

### 2.5 Two-Dimensional Profile Likelihood as a Design Criterion

**FIGURE 1:**
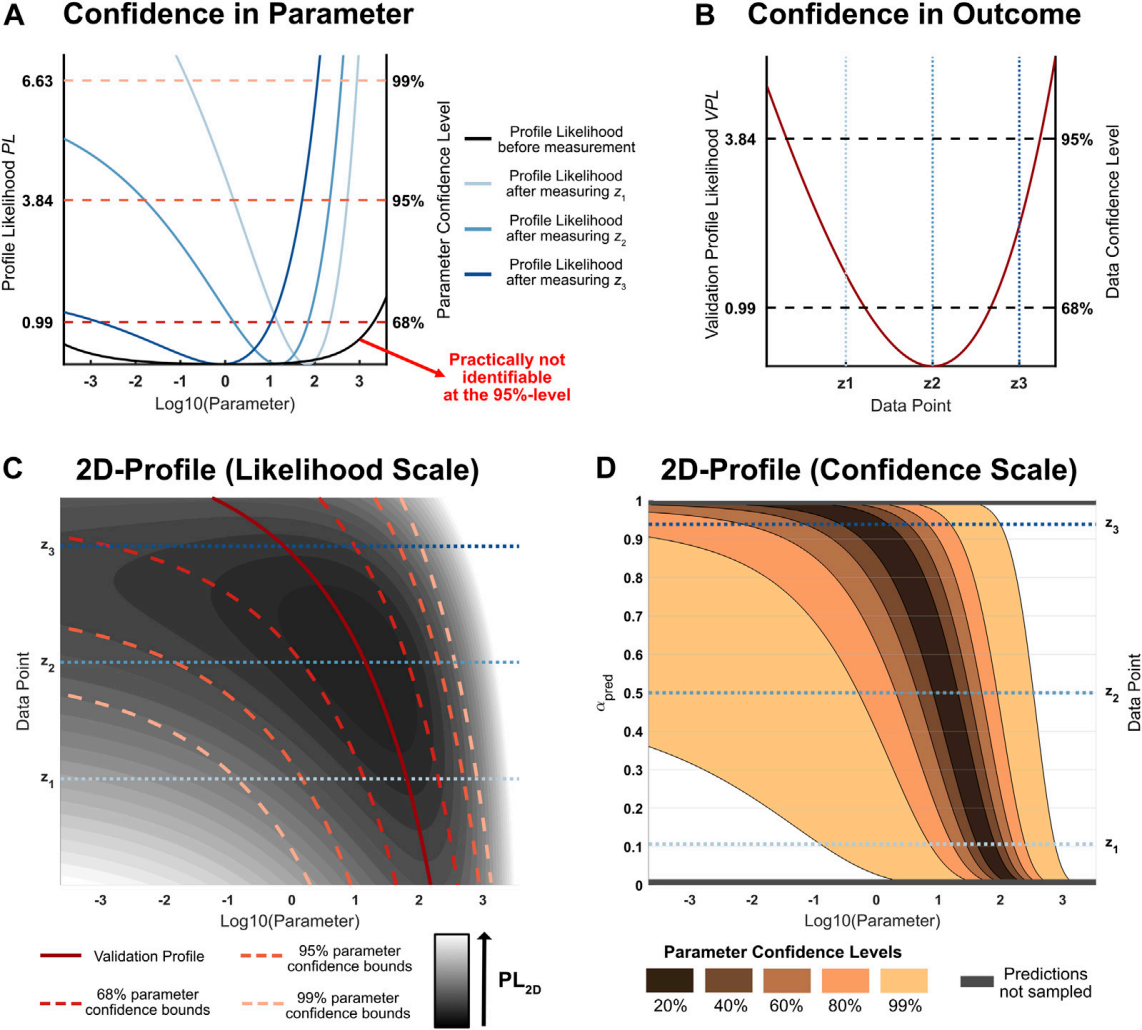
**(A)**: Likelihood profiles of a hypothetical parameter of interest. Different measurement outcomes *z*_1_, *z*_2_, *z*_3_ for the same experimental condition lead to different updated parameter profiles which assess uncertainty about a parameter of interest. **(B)**: Validation profile of the considered hypothetical experimental condition. This profile assesses the likelihood of a new measurement: The smaller the validation profile value, the more likely the respective outcome **(C)**: 2D-Likelihood profile for the parameter of interest under some given experimental condition. The vertical axis corresponds to different possible measurement outcomes. If the outcome on the vertical axis would be observed, the profile likelihood after the measurement is given by the corresponding horizontal cross-section through the two-dimensional profile. In this example, lower values of the measurement outcome lead to narrow parameter confidence intervals after the measurement. **(D)**: 2D-Likelihood profile on a confidence scale. Intervals of the same size on the *y*-axis hold equal confidence that a measurement will yield a data point in the corresponding interval. The prediction confidence levels on the vertical axis illustrate that the sampled two-dimensional likelihood profile covers most of the plausible measurement outcomes.

In the previous sections, we proposed to quantify parameter uncertainty via the profile likelihood approach by definition of an average profile width in Eq. 8, which summarizes the existing uncertainty about the parameter of interest. Optimal experimental design aims at minimizing this measure of uncertainty by choosing an experimental condition for the next measurement which optimizes a suitable design criterion. However, for a given experimental condition *D* it is a priori unknown which value will result from a future measurement. This implies that the parameter profile likelihood $PL_{p_{i}}\left(\beta |Y,z\right)$ and therefore the average profile width $\bar{w}\left(p_{i}|Y,z\right)$ depends on the measurement outcomes *z*. In Figure 1A, the original parameter profile before the measurement (black line) is practically non-identifiable at the 95%-level for the parameter of interest. Depending on the measured data point *z*_*i*_, the uncertainty about the parameter of interest is reduced to different degrees as indicated by the corresponding parameter profiles (blue lines). Since the measurement outcome is unknown, it is unclear what the uncertainty will be after the measurement.

The plausibility of different possible measurement outcomes can be accounted for by weighting the average profile widths for different measurement outcomes by their likelihood of occurrence. As discussed in Section 2.2.2, this plausibility measure is implied by the validation profile likelihood. Figure 1B shows the validation profile for the specified design: *z*_2_ corresponds to the current maximum likelihood prediction for this experimental condition and is therefore the most likely measurement outcome given the current evidence, while *z*_3_ has a higher validation profile value than *z*_1_ and is therefore less likely. Therefore, the validation profile likelihood implies a predictive distribution which can be defined in analogy to the confidence distribution derived from the parameter profile likelihood. The corresponding predictive density *ρ* ^*pred*^(*z*|*Y*) associates different measurement outcomes with our confidence that the specific outcomes occur.

The concept of summarizing parameter uncertainty for a fixed measurement outcome and subsequent aggregation of different possible measurement outcomes based on the predictive density can be combined to construct a design criterion for an experimental condition of interest. To this end, each expected parameter profile width $\bar{w}\left(p_{i}|Y,z\right)$ is weighted with the certainty *ρ* ^*pred*^(*z*|*Y*) of observing measurement outcome *z* and the expected average profile width follows as

$$W\left(p_{i}|Y,D\right)=\int \bar{w}\left(p_{i}|Y,z\right)\rho^{pred}\left(z|Y\right)dz.$$ (9)

*W*(*p*_*i*_|*Y*, *D*) exclusively depends on the given data *Y* and the experimental condition *D* of a subsequent experiment and thus by definition constitutes a design criterion. This quantity can be interpreted as the expected average profile width after measuring at the experimental condition *D*, where the average is taken over different parameter confidence levels and the expectation is taken over different possible measurement outcomes, weighted by their predicted plausibility. Given a set of experimental conditions {*D*_1_, …, *D*_*n*_}, the optimal experiment D* to inform parameter *p*_*i*_ is the one which minimizes *W*(*p*_*i*_|*Y*, *D*) given the current data *Y*, i.e.

$$D^{*}=\underset{D\in \left\{D_{1},\ldots ,D_{n}\right\}}{\mathrm{arg min}}W\left(p_{i}|Y,D\right).$$ (10)

The information necessary to evaluate the design criterion in Eq. 9 is summarized by a two-dimensional likelihood profile, defined as

$$PL_{2D}\left(z,\beta |D_{z}\right)≔-2\ln\left(\frac{L\left(\beta ,\hat{\omega }\left(\beta |Y,z\right)|Y,z\right)}{L\left(\hat{\theta }\left(Y\right)|Y,F\left(\hat{\theta }\left(Y\right)|D_{z}\right)\right)}\right).$$ (11)

For any fixed measurement outcome *z*, the resulting parameter profile likelihood can be extracted from this quantity. Simultaneously, the two-dimensional likelihood profile contains information about the plausibility of different measurement outcomes. Figure 1C illustrates this relationship: The profiles in Figure 1A are horizontal cross-sections from the two-dimensional likelihood profile (blue lines). The minimal profile value of each horizontal cross-section defines the path of the validation profile in Figure 1B (solid red line). The confidence intervals (dashed red lines) depend on the different possible measurement outcomes: Some measurement outcomes lead to more information about the parameter of interest than others as indicated by narrower confidence intervals.

The process of averaging confidence interval widths over the various parameter confidence levels and taking the expected value over the possible plausible data realizations is visualized in Figure 1D. The displayed two-dimensional profile is based on the same data as depicted in Figure 1C, but it has been transformed onto a different scale. The minimum of each horizontal cross section is shifted to the common null value, but still represents the trajectory of the validation profile. For the transformed two-dimensional likelihood profile, the vertical axis is proportional to the prediction confidence levels, i.e. intervals with the same length correspond to an equal confidence of yielding a measurement value in the given intervals. This transformation reveals that the interval [*z*_1_, *z*_3_] is a 83*%*-prediction interval for a future measurement outcome given the experimental condition. The horizontal gray patches at the top and the bottom of Figure 1D correspond to all the measurement outcomes for which the original two-dimensional profile likelihood was not sampled, because they are unlikely to occur. The trend of different parameter confidence intervals as a function of different data points is illustrated for five discrete confidence levels (shades of red). On this scale, the expected average profile width *W*(*β*|*Y*, *D*) is equal to the average of all the colored areas, where the smaller confidence intervals are included in the larger ones.

### 2.6 Experimental Design Workflow

**FIGURE 2:**
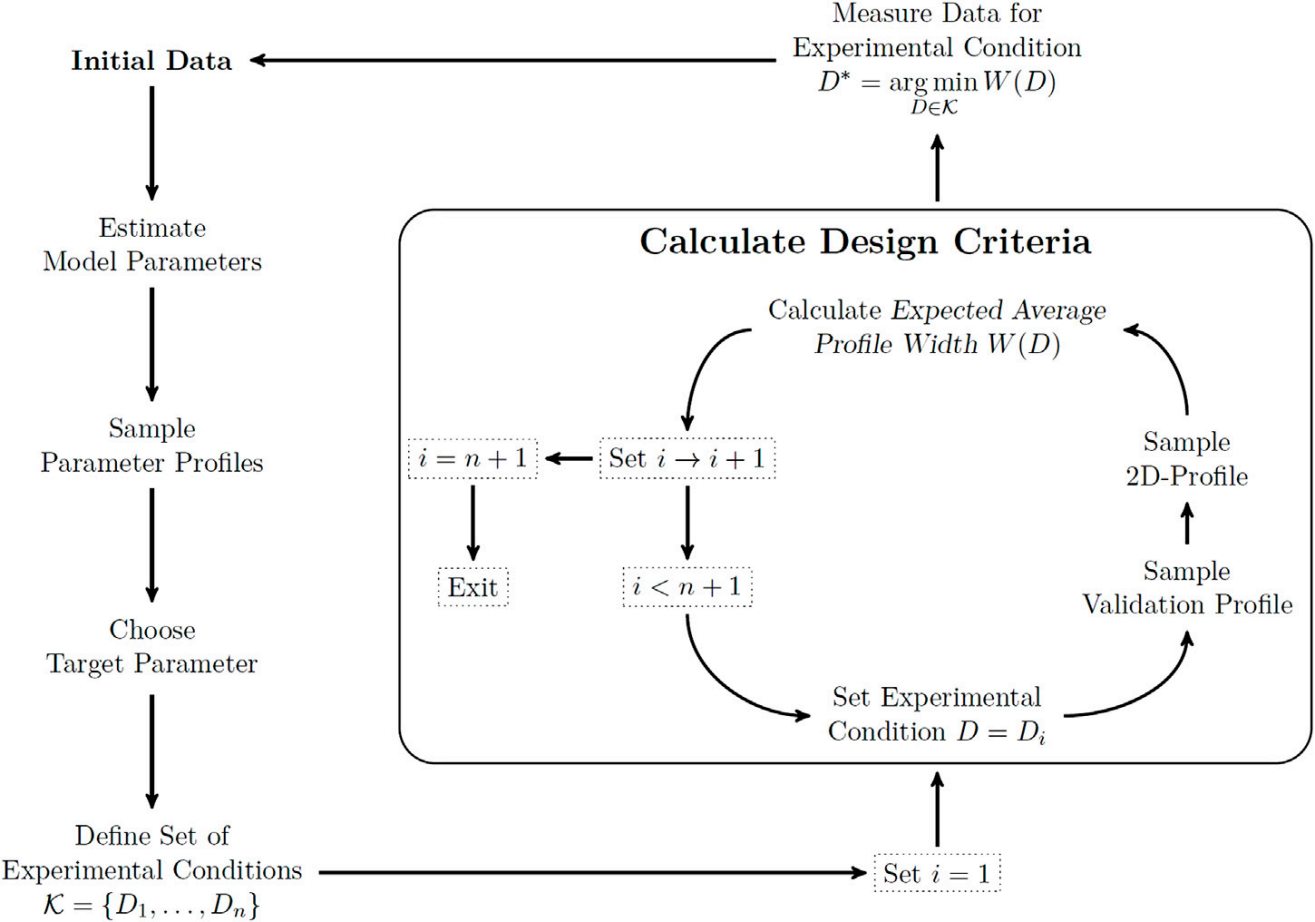
Workflow for the sequential experimental design scheme. Starting from the current data set (top left), the target parameter is chosen and relevant experimental conditions are specified. Calculating the two-dimensional profile likelihood and evaluating the expected average profile width for each experimental condition (box) reveals the optimal condition for the next measurement. Dotted rectangles specify the state of the *for loop*, while text without rectangles correspond to the experimental design steps involved.

Utilization of two-dimensional likelihood profiles as a tool for experimental design requires a ready-to-use workflow in applications. We provide an example for this workflow in a fully sequential experimental design scheme to put the previous definitions into a more practical context. Figure 2 shows a flowchart of the steps involved in this workflow. Starting from an initial data set, the model parameters are estimated and the profile likelihood is calculated for all model parameters to obtain information about existing parameter uncertainties. The likelihood profiles are calculated by numerical evaluation of Eq. 4 for a finite set of profile parameters. If there are non-identifiable parameters, the biologically most relevant parameter is targeted for improvement by the experimental design scheme.

After a representative set of experimental conditions has been defined, the design criterion in Eq. 9 needs to be evaluated for each of the experimental conditions by the following steps. First, a validation profile is calculated for the experimental condition. This validation profile provides the range of relevant measurement outcomes for the respective experimental condition. Therefore, the space on which the two-dimensional likelihood profile needs to be sampled is finite. This space is sampled by evaluating the parameter profile likelihood for a representative set of measurement outcomes. The expected average profile width is calculated from the two-dimensional likelihood profile by employing the discrete counterparts of all expressions appearing in Eq. 9. At this point the details are more of technical than conceptual relevance and we want to emphasize that an automated implementation of this algorithm is available and referred to at the end of this manuscript. The final step of the workflow is now to choose the experimental condition which provides the minimal value for the design criterion as the target for the next measurement. This workflow can be repeated after a new data point has been generated to determine a sequence of informative measurements.

## 3 Results

We illustrate the process of choosing the best experimental design for a parameter of interest by two examples. The first example is based on simulated data for a simple model with two consecutive reactions in which compound A is converted to compound B which is then converted to compound C and is therefore termed as ABC model in the following. This example will serve to illustrate the interpretation of the two-dimensional profile likelihood. The second example is based on the published experimental data for a model of erythropoietin (EPO) degradation (Becker et al., 2010) for which data has been censored in order to mimic a setting in which experimental design can be applied. This example serves to explain the full workflow of the sequential experimental design scheme in an application setting and illustrates the practical feasibility of our approach.

### 3.1 Experimental Design in the ABC-Model

**FIGURE 3:**
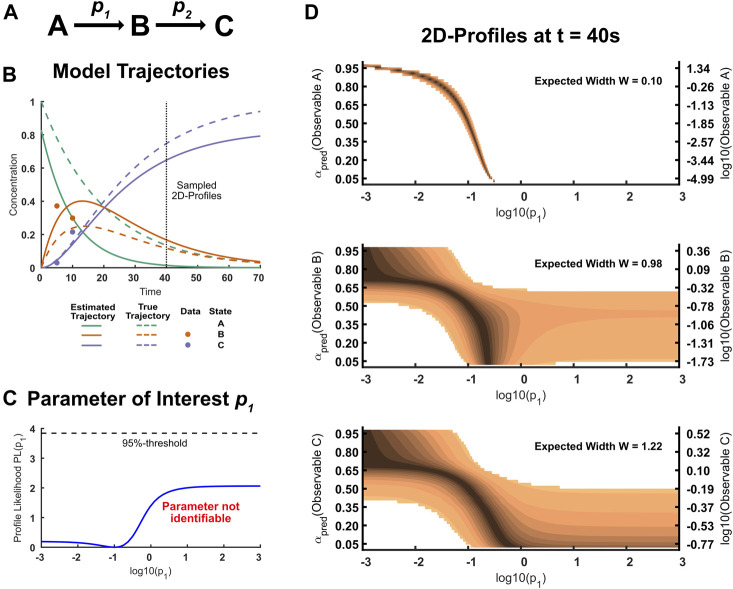
**(A)**: States and parameters in the ABC-model. The model has three parameters: Two rate constants *p*_1_ and *p*_2_, and the initial concentration *A*_0_. The initial concentrations of B and C are assumed to be zero. **(B)**: Trajectories of the ABC-Model. The dots correspond to the sparse data simulated from the true model. In this example, state B and C were assumed to be observable, but have only been observed at early time points. The true trajectory of state A yet differs considerably from the estimated trajectory. **(C)**: Likelihood profile of the practically non-identifiable parameter *p_1_*. Because the initial concentration of state A is unknown, this parameter is difficult to estimate without information about state A. **(D)**: 2D-Likelihood profiles for the three states A, B and C if measured at time point t = 40. The illustrated profiles are presented on a confidence scale according to Figure 1D. If state A was observable, the finite width of the 2D-profile to the 95% level indicates that any measurement outcome will make the parameter *p*_1_ identifiable. Note that possible values for A scatter across six orders of magnitude because the predictions for A are barely informed. Measuring state B or C will likely put an lower or upper limit on the parameter *p*_1_.

The ABC model describes a simple case of a model in which the model predictions non-linearly depend on the model parameters. The reactions are illustrated in Figure 3A: State A is converted to state B with the rate *p*_1_ and B is subsequently converted into compound C with rate *p*_2_. In a biochemical setting, these three states might represent three conformations of three activation states in terms of different phosphorylations. The dynamics of this system are determined by the following differential equations:

$$\begin{array}{ll} \dot{A} & =-p_{1}A\\ \dot{B} & =p_{1}A-p_{2}B\\ \dot{C} & =p_{2}B. \end{array}$$

In order to illustrate the two-dimensional likelihood profile approach on a simple model for which we know the true underlying parameters, we defined the true model parameters and simulated data from this model. In this true model, the initial concentrations of state B and state C were set to zero which was assumed to be known for inference, such that the system is characterized by the three parameters {*p*_1_, *p*_2_, *A*_0_} which were assumed to be unknown and have to be estimated from data. The data set simulated from the true model parameters is sparse: state A has been assumed to not be observable and the two data points available for each state B and C have been generated with an initial concentration log(*A*_0_) = 0 from log-normal distributions with a standard deviation *σ*_log_ = 0.2 After simulation of the data, the model parameters {*p*_1_, *p*_2_, *A*_0_} are optimized to estimate their values and the corresponding state trajectories.

The true as well as the estimated state trajectories are illustrated in Figure 3B: While the model predictions fit the data well, there is still considerable disagreement of the underlying true model and the best model fit. This is especially true for state A, considering that the differences between trajectories are analyzed on a log-scale, which measures relative differences. An analysis of parameter uncertainty reveals that parameter *p*_2_ is identifiable, as information for state B and C suffices to inform this rate. By similar reasoning, there is less information available for parameter *p*_1_ and the corresponding profile likelihood reveals that the parameter is practically non-identifiable over the whole considered parameter space, as illustrated in Figure 3C.

In our example, we want to inform this practically non-identifiable parameter *p*_1_ by choosing a measurement out of three possible experimental conditions. For demonstration purposes, the three experimental conditions of measuring either state A, B or C at the time point *t* = 40 are considered. The corresponding two-dimensional likelihood profiles are illustrated in Figure 3D. If it was possible to measure observable A, this would be highly informative and in fact guarantees that the parameter *p*_1_ is identifiable no matter the outcome of the measurement. This is intuitive, since the measurement of the yet unobserved quantity A highly constrains the possible dynamics. It should be noted that possible outcomes for the observable A vary across orders of magnitudes which can be attributed to the fact that the dynamics for A are poorly constrained given the current data set. The two-dimensional profiles associated with observable B and C reveal that the parameter *p*_1_ will likely not be identifiable even after the measurement. However, the magnitude of outcomes will yield at least an upper or a lower bound for the parameter of interest: Large values of B and C put an upper limit on *p*_1_, as this means that the reaction can not be arbitrarily fast, while low values of B and C put a lower limit of *p*_1_ because the reaction can not be arbitrarily slow. A not immediately obvious result from the two-dimensional profiles is that measuring observable B is more informative than measuring observable C as seen from the calculated design criterion. This example illustrates that two-dimensional likelihood profiles provide qualitative as well as quantitative information about how experiments impact parameter uncertainty.

### 3.2 Experimental Design in the Erythropoietin Degradation Model

The modeled system for the degradation of erythropoietin (EPO) (Becker et al., 2010) is an example of a non-linear model with intertwined reactions of biochemical states. EPO acts as a ligand by binding to the corresponding cell receptor to form a complex. This complex is internalized and then EPO is degraded. The mathematical model provided the insight that a combination of EPO receptor turnover and recycling guarantees that biochemical response to a broad range of ligand concentrations is possible (Becker et al., 2010).

**FIGURE 4:**
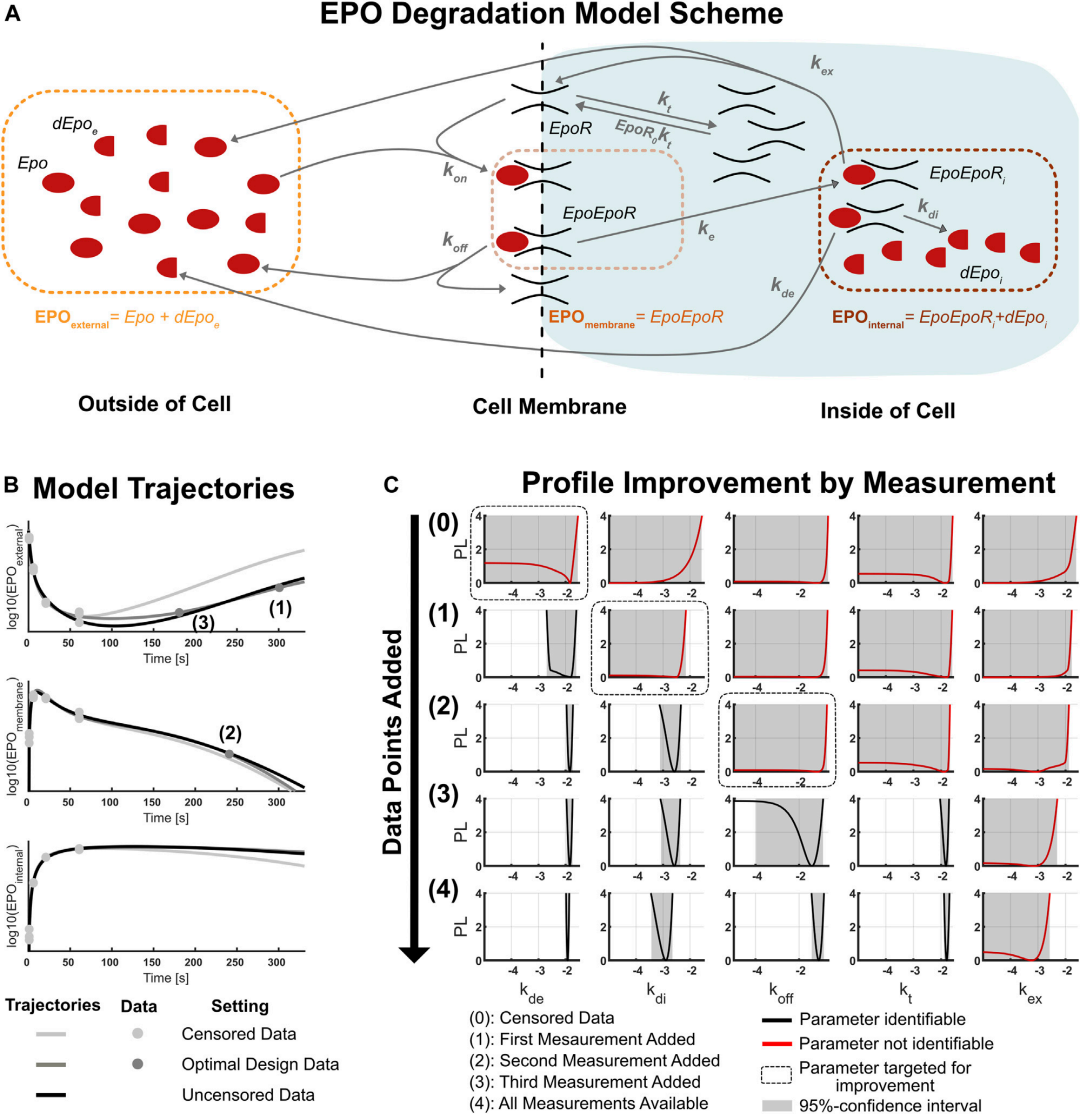
**(A)**: Scheme of the biological dynamics in the EPO degradation model (Becker et al., 2010). There are six model states (black text) which interact through different biological reactions (gray arrows) and three observables (colored text). EPO is transported into the cell and degraded there. **(B)**: Model trajectories for the observables of the EPO-model. The plotted curves are the best fit trajectories for three different data sets: the censored data set used at the start of the experimental design analysis, the data set after adding three sequentially proposed data points, and the uncensored published data set. The numbers indicate the order of the sequentially measured data points. **(C)**: Change of parameter likelihood profiles during the sequential experimental design procedure. The targeted parameter always became identifiable after data for the optimal experimental condition proposed by the two-dimensional likelihood approach was added into the model. Incorporating the three optimal data points into the model already produces results of similar accuracy compared to the published data set with 36 additional data points.

A scheme of reactions in the biological system is illustrated in Figure 4A. The model features six dynamic states: EPO (*Epo*) and degraded EPO (*dEpo*_*e*_) outside of the cell, EPO receptors (*EpoR*) and EPO–EPO receptor complexes (*EpoEpoR*) on the cell membrane, and internalized EPO–EPO receptor complexes (*EpoEpoR*_*i*_) and degraded EPO (*dEpo*_*i*_) inside of the cell. The reactions illustrated in the figure can be translated into the following set of differential equations (Becker et al., 2010):

$$\begin{array}{ll} \frac{d}{dt}EpoR & =k_{t}\cdot \left(EpoR_{0}-EpoR\right)-k_{on}\cdot Epo\cdot EpoR+k_{o\mathrm{ff}}\cdot EpoEpoR+k_{ex}\cdot EpoEpoR_{i}\\ \frac{d}{dt}Epo & =-k_{on}\cdot Epo\cdot EpoR+k_{o\mathrm{ff}}\cdot EpoEpoR+k_{ex}\cdot EpoEpoR_{i}\\ \frac{d}{dt}EpoEpoR & =k_{on}\cdot Epo\cdot EpoR-k_{o\mathrm{ff}}\cdot EpoEpoR-k_{e}\cdot EpoEpoR\\ \frac{d}{dt}EpoEpoR_{i} & =k_{e}\cdot EpoEpoR-k_{de}\cdot EpoEpoR_{i}-k_{di}\cdot EpoEpoR_{i}-k_{ex}\cdot EpoEpoR_{i}\\ \frac{d}{dt}dEpo_{i} & =k_{di}\cdot EpoEpoR_{i}\\ \frac{d}{dt}dEpo_{e} & =k_{de}\cdot EpoEpoR_{i}. \end{array}$$

There are seven dynamic parameters (*k*_*t*_, *k*_*on*_, *k*_*off*_, *k*_*ex*_, *k*_*e*_, *k*_*de*_, *k*_*di*_) and two unknown initial conditions (*Epo*_0_, *EpoR*_0_) in the model which are biologically interpretable as well as six further parameters which appear only in the observation function and not in the dynamic model. Because EPO can be traced with a radioactive marker, the concentration of EPO can be measured outside of the cell (*EPO*_*external*_), on the cell membrane (*EPO*_*membrane*_) and inside of the cell (*EPO*_*internal*_). This provides us with three different observables for the six dynamic states, i.e. the model is only partially observable.

The parameters of the model are identifiable except for one parameter given the complete data set of the study. In order to illustrate experimental design considerations, a model which is not yet well informed by data is required. Thus, we censored one half of the complete data set for all observables which respectively correspond to the later stages of the dynamics. This serves two purposes: First, we reduced the information content of the data, thus creating non-identifiabilities for some parameters. Second, we gain access to biological data for 3 (observables) x 4 (time points) = 12 experimental conditions which can be used to mimic real measurements.

The best fit model trajectories for the three observables are illustrated in Figure 4B for three sets of data (shades of gray). One set of trajectories corresponds to the censored data set, the next set corresponds to an optimal sequential experimental design with three additional measurements and the last set of trajectories corresponds to the original full data set with 3 (observables) x 4 (time points) x 3 (replications) = 36 additional data points. The predictions change significantly when adding the three optimal data points to the censored data set, while adding the rest of the data only changes the model trajectories slightly.

The three optimal data points were determined by applying the workflow for the sequential experimental design scheme shown in Figure 2. The iterative improvement of the likelihood profiles of the non-identifiable parameters by this workflow is illustrated in Figure 4C. Starting with the censored data set, five parameters are non-identifiable. This comprises the external and internal EPO degradation rate *k*_*de*_ and *k*_*di*_, the complex dissociation constant *k*_*off*_, the receptor turnover rate *k*_*t*_ and the complex recycling rate *k*_*ex*_.

The internal EPO degradation rate *k*_*de*_ was targeted by the first experiment, and has been made identifiable after measuring *EPO*_*external*_ at a late stage of the dynamics. Note that retrospectively, this choice was highly tailored to the identification of *k*_*de*_, as the profile likelihood for the other parameters only changed slightly. This underlines that experiments proposed by our approach aim specifically at improving the knowledge about the targeted parameter of interest.

In the second experiment, the internal EPO degradation rate *k*_*di*_ was targeted. The corresponding optimal experiment is a measurement of *EPO*_*membrane*_ at a late time point. Because this design is optimal at an earlier time point than the first measurement, this suggests that the first measurement of *EPO*_*external*_ already carries information which could have been obtained from measuring *EPO*_*membrane*_ at the same time point, highlighting that model dynamics are highly intertwined. Imitating the measurement for the proposed experimental design again shows that the targeted parameter is identifiable after the experiment, while the others are still practically non-identifiable.

The third iteration of experimental design targeted the complex dissociation constant *k*_*off*_ and revealed that measuring *EPO*_*external*_ at an earlier time point is now more informative than measuring the observable *EPO*_*internal*_, for which late time measurements are still not available. This highlights the fact that determination of the optimal experimental design is difficult by intuitive considerations and experimental design approaches provide non-trivial insights. This measurement removed the non-identifiability of both the targeted parameter *k*_*off*_ and also the turnover rate *k*_*t*_ which was not considered when planning the experiment.

A fourth iteration of the sequential experimental design was not conducted because the two-dimensional likelihood profiles for the last non-identifiable parameter *k*_*ex*_ indicate that a single additional data point for any of the remaining experimental conditions does not provide enough information to make the parameter identifiable. In fact, this is in line with the results of the final model with all data available, as the parameter is still practically non-identifiable given the complete data set. The two-dimensional likelihood profiles corresponding to the four experimental design iterations are illustrated in the Supplementary Figures S1–S4.

The comparison of parameter likelihood profiles for the design with three optimally chosen measurements with the full data set design of 36 new data points is shown in the last two rows of Figure 4C. The similarity of all profiles across all parameters indicates that three optimally chosen experimental conditions already yield much of the information contained in the set of all 36 data points. This underlines the ability of optimal experimental design to reduce the amount of data needed to remove non-identifiabilities for the parameters of interest. Therefore, application of the optimal sequential experimental design on a realistic biological model demonstrated the feasibility and merits of the two-dimensional likelihood profiles as an approach for experimental design.

## 4 Discussion

### 4.1 Experimental Design by Two-Dimensional Likelihood Profiles

A well-planned experiment can save time and resources. Therefore, optimal experimental design aimed at reducing the amount of data needed to inform the model is desirable in any context, but this task is often non-trivial for complex models such as those encountered in systems biology. We established a method for optimal experimental design aiming at reducing parameter uncertainty for a single parameter of interest in a frequentist setting. To this end, we define two-dimensional likelihood profiles which contain information about the likely parameter uncertainty after a measurement. Our approach for experimental design employs the theoretically appealing concept of likelihood profiles, which can serve as a measure for uncertainty in parameter estimates but also for a measure of uncertainty of measurement outcomes. These measures can be conceptually understood to imply confidence densities for parameters or predictive densities for measurement outcomes with strictly frequentist concepts. The presented approach allows for the evaluation of the impact of an experiment in a qualitative as well as in a quantitative manner.

The two-dimensional profile likelihood approach for experimental design was employed in two examples to illustrate its properties and establish feasibility of the method. The ABC reaction model features a non-linear relationship between model states and parameters and served to illustrate the features of two-dimensional likelihood profiles. In order to show practical feasibility of the approach in a realistic setting, an established erythropoietin degradation model (Becker et al., 2010) was investigated. To this end, half of the full data set has been censored to simulate a realistic setting for experimental design in which some model parameters were practically non-identifiable. A fully sequential experimental design procedure indicated that only 3 of the 36 censored data points were required to successfully remove all possible parameter non-identifiabilities.

### 4.2 Implementation and Limitations

The numerical implementation is provided as part of the *Data2Dynamics* (Steiert et al., 2019) modeling environment in *MATLAB*. The algorithm exploits the existing one dimensional profile likelihood calculation in order to construct the two-dimensional profile likelihood. Computationally, this amounts to about ∼1,000 local optimizations per two-dimensional likelihood profile, where local optimization is to be understood as deterministic optimization from a good initial guess for the parameters. Robustness of these fits is generally easier to obtain if the available data is appropriate for the size of the model, such that the model dynamics are constrained to some degree.

Problems associated with limited data availability go beyond numerical issues and are rooted in the structure of our approach. As a frequentist method, all information used in our experimental design scheme must stem from the data already measured. We have not assessed how much data needs to be initially available before a systematic experimental design procedure is practically feasible. However, the issue of lacking prior knowledge is not exclusive to our approach and a more general theme in non-linear experimental design. For the application in systems biology, initial data is often needed in proposing a suitable model, such that there will usually be data to start off with.

A practical limitation induced by insufficient data occurs if the range of reasonable measurement outcomes can not be predicted by the model, i.e. the validation profile reveals a practical non-identifiability of the model prediction. The existence of this non-identifiability complicates the estimation of the expected parameter uncertainty in the two-dimensional profile likelihood approach, because it relies on the prediction of the measurement outcomes given the model and current data. On the one hand, this fully utilizes the information available in the model, but on the other hand this constrains the applicability of the approach if the prediction for the measurement outcome is insufficiently constrained by the available model data. In case the model prediction of interest is not identifiable, a weak quadratic prior can be added to the validation profile in order to guarantee a finite sample space. This heuristic approach increases the scope of possible application settings. We emphasize that our experimental design procedure works best from a computational as well as methodical point of view if enough data is available such that model predictions are at least loosely constrained.

The usual assumption of the correctness of the model structure is especially important in our proposed method because it utilizes the model for predicting likely outcomes of the experiment and for calculating existing parameter uncertainties. This assumption is usually implicitly contained in any design strategy, but we emphasize that the full exploitation of the likelihood in our approach implies that the proposed experimental design will benefit greatly from solid prior knowledge about the model structure. This does not apply to prior knowledge about model parameters, because likelihood profiles account for parameter uncertainties.

The relationship between confidence intervals and likelihood profiles critically depends on the distributional assumption for the corresponding likelihood profile in Eq. 6. The implicit assumption that these likelihood ratios are $\chi_{1}^{2}$ distributed for the true parameter set in general holds only asymptotically. However, as this is general practice in the interpretation of likelihood profiles, we follow this procedure and underline that improving upon this assumption offers opportunities for improving the assessment of parameter uncertainties.

### 4.3 Comparison to Existing Methods

There are two conceptually different methods in the literature which we want to discuss, neglecting approaches based on the Fisher information matrix as reasoned before. One branch of methods deals with a Bayesian approach to experimental design which utilizes the Shannon information of the posterior distribution to plan optimal experiments. The other branch of methods discusses the concepts of frequentist approaches which find experimental designs by sampling relevant regions of the parameter space in order to assess the sensitivity of model predictions with respect to these parameters.

The Bayesian approach (Busetto et al., 2013; Huan and Marzouk, 2013; Liepe et al., 2013) is conceptually similar to our approach, but only applicable if suitable prior parameter distributions are available. The posterior parameter distribution after a possible measurement depends on unknown measurement outcomes which can be resolved by averaging the posterior distribution over the Bayesian predictive density. Similarly, our proposed frequentist method utilizes a predictive density for the measurement outcomes and a confidence density for the parameter estimates, eliminating the need for prior distributions. These “distributional estimators” (Xie and Singh, 2013) are implicitly derived from the likelihood profiles. This theoretical framework suggests the use of confidence and predictive densities in quantifying the confidence that an interval of parameter values or measurement outcomes contains the true parameter value or, respectively, a future measurement outcome.

Our method explicitly determines the impact of different plausible measurement outcomes of an experimental design on the parameter estimate of interest in order to derive a design criterion. This is different to existing frequentist approaches (Bazil et al., 2012; Steiert et al., 2012) which consider the sensitivity of model predictions to the different parameters which are consistent with the current data. Predictions which largely vary under these acceptable parameters indicate experimental conditions which are likely informative as they constrain the set of possible model dynamics. This approach has been awarded as best performing in the DREAM6 challenge (Steiert et al., 2012), although the feedback of the possible measurement results on the model parameter is not considered directly. This hinders intuitive interpretation of how a possible experiment feeds back into the parameter of interest and lacks a quantitative assessment of what constitutes a large variation of model predictions. Reversing the logic of this approach by considering the impact of likely model predictions on the parameter of interest leads to our refined approach, although this requires a higher computational cost.

### 4.4 Implications for Research

Our proposed approach can be used to select the most informative experimental design for a targeted parameter of interest. This is often relevant if there are certain biological parameters of interest which are not identifiable given the current data. We want to emphasize that although we discussed reduction of uncertainty for a single target parameter of interest, generalization to reducing the uncertainty for a model prediction, i.e. for a function of model parameters, is straightforward. The detailed quantitative and qualitative information gain by comparing two-dimensional profiles for the different experimental conditions comes with a higher computational cost compared to other approaches. As such, the detailed information provided by our method might be especially useful if experimental measurements require considerable time and resources and as such accuracy is favored over computational efficiency.

The experimental design approach only requires the existence of a suitable likelihood function and is therefore applicable in a broad spectrum of applications. We emphasize the novelty of our approach in employing confidence and predictive distributions as frequentist distributional measures for the confidence in parameter and measurement outcomes, which serve a similar function as Bayesian probabilities. Exploring the interaction of these concepts provides a point of interest for further research in frequentist experimental design.

## 4.5 Conclusion

To summarize, we established an experimental design procedure which aims at reducing the uncertainty for a parameter of interest. This design procedure reduces the likelihood function to a two-dimensional likelihood profile: One dimension informs our confidence of observing a certain measurement outcome for the given experimental condition, while the other dimension informs our confidence in the model parameter corresponding to the underlying true parameter. Testing our experimental design procedure on a simple model with simulated data and on a real model with experimental data revealed that our approach accurately predicted relevant experimental designs. Our method provides detailed information about possible experimental conditions on an easily interpretable quantitative as well as qualitative level.

## Data Availability

The datasets presented in this study can be found in online repositories. The names of the repository/repositories and accession number(s) can be found below: doi.org/10.6084/m9.figshare.16863559, https://github.com/Data2Dynamics/d2d.
