# Supplementary material for: Optimal Experimental Design Based on Two-Dimensional Likelihood Profiles
Source: Front Mol Biosci. 2022 Feb 23;9:800856. doi: 10.3389/fmolb.2022.800856 (PMC8906444; doi:10.3389/fmolb.2022.800856)
Supplement: Supplementary file 1 [file DataSheet1.PDF]

Supplementary Material

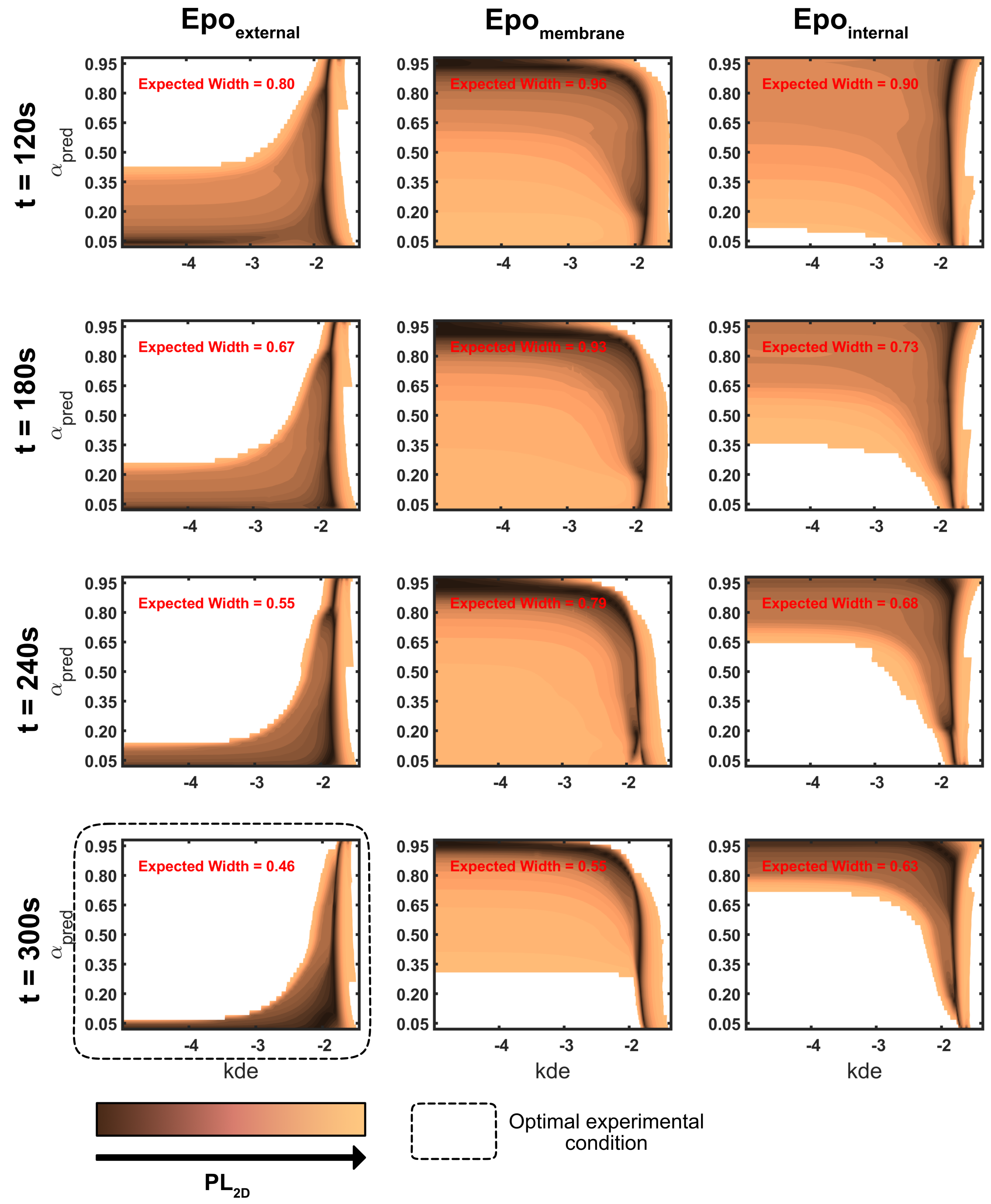

Figure S1. Two-dimensional likelihood profiles for the censored EPO-degradation model for parameter k_{de}. The different panels correspond to different measurement times (vertical axis) and different observables (horizontal axis). The vertical axis for the individual 2D-profiles indicates prediction confidence levels as in Figure 1D. The optimal experiment is measuring $EPO_{external}$ at $t = 300s$.

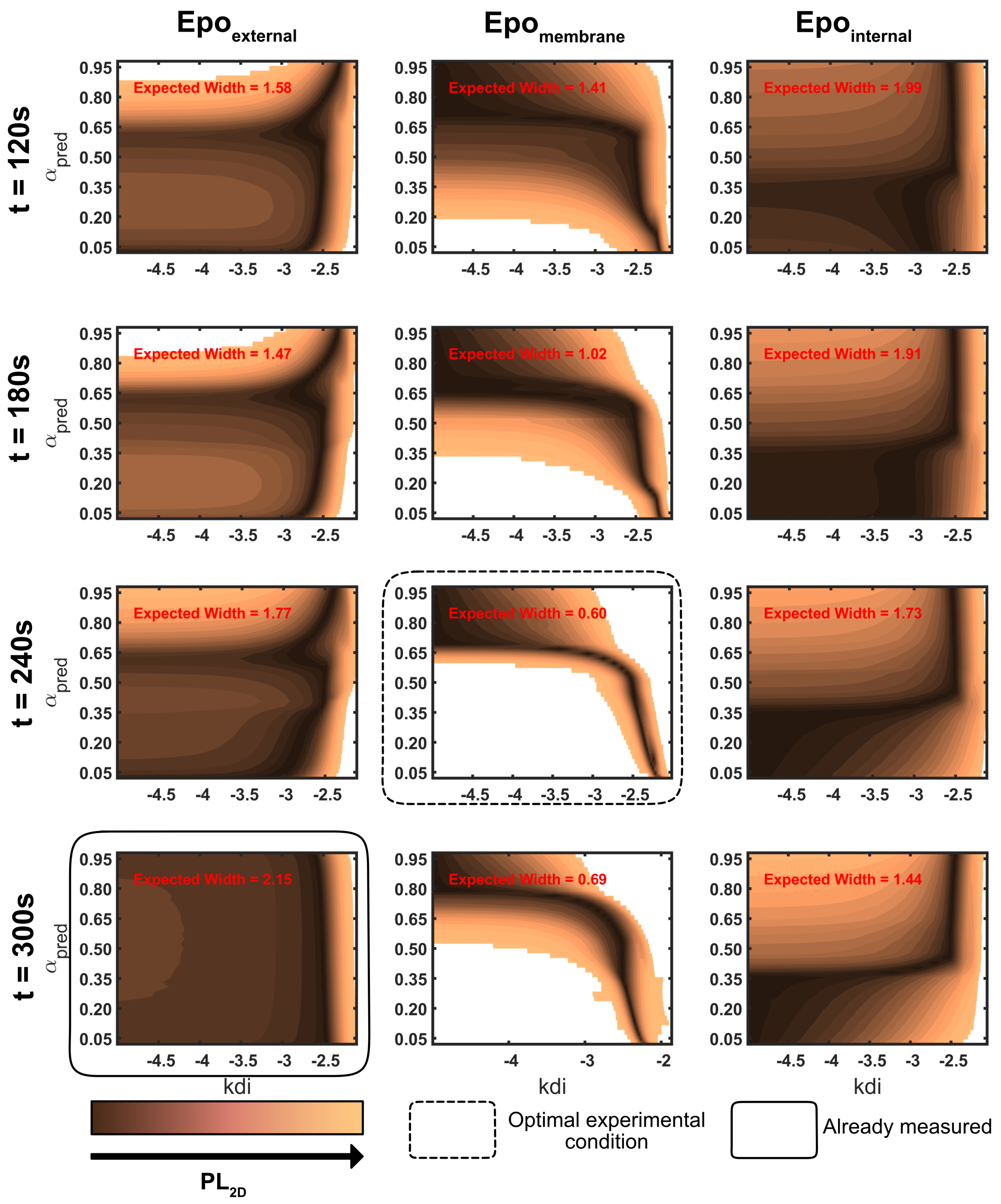

Figure S2. Two-dimensional likelihood profiles for the censored EPO-degradation model for parameter k_{di}. The different panels correspond to different measurement times (vertical axis) and different observables (horizontal axis). The vertical axis for the individual 2D-profiles indicates prediction confidence levels as in Figure 1D. The optimal experiment is measuring $EPO_{membrane}$ at $t = 240s$. Measuring at a time point for which data exists is uninformative compared to measuring at other time points.

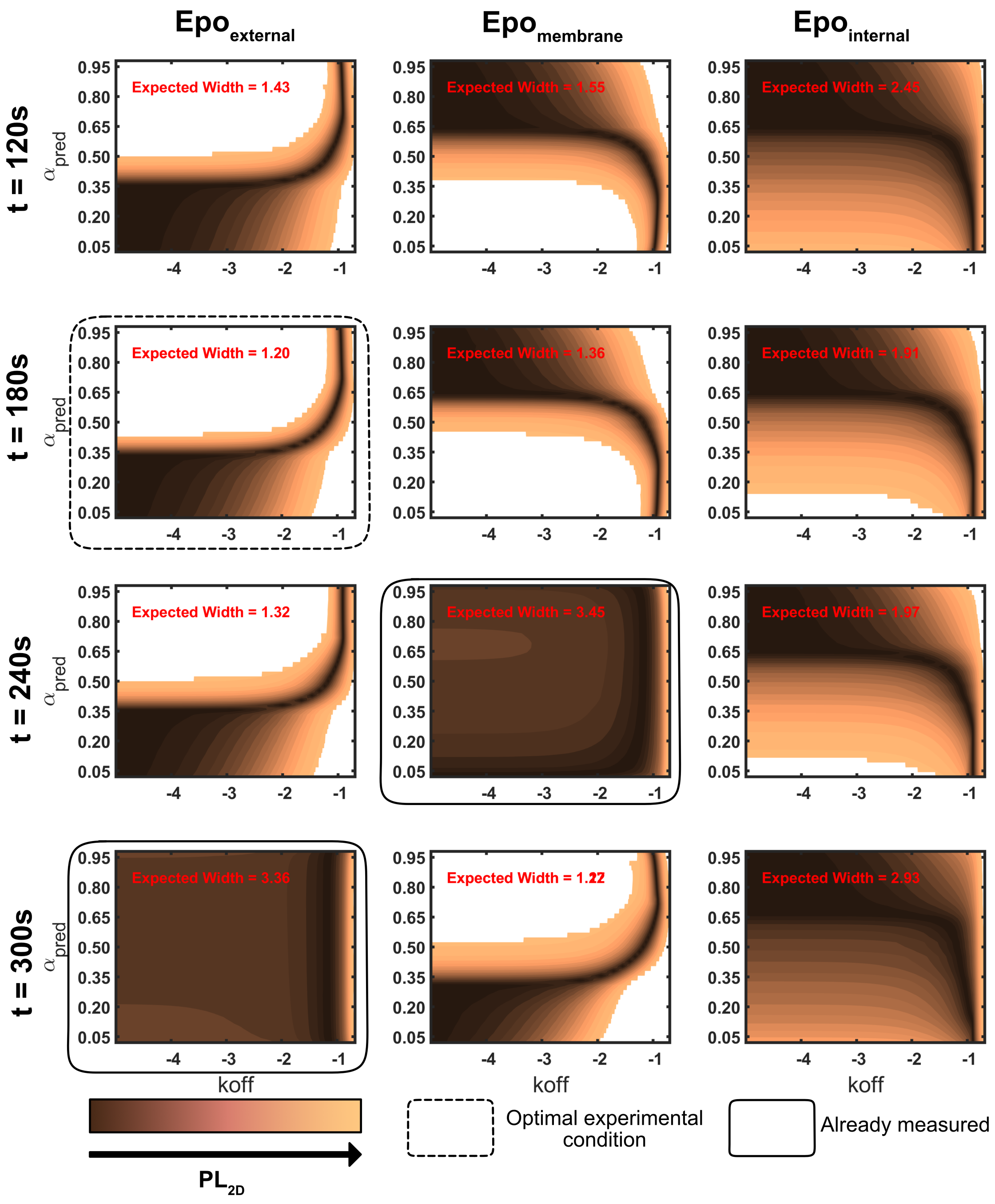

Figure S3. Two-dimensional likelihood profiles for the censored EPO-degradation model for parameter k_{off}. The different panels correspond to different measurement times (vertical axis) and different observables (horizontal axis). The vertical axis for the individual 2D-profiles indicates prediction confidence levels as in Figure 1D. The optimal experiment is measuring $EPO_{external}$ at $t = 180s$. Measuring at a time point for which data exists is uninformative compared to measuring at other time points.

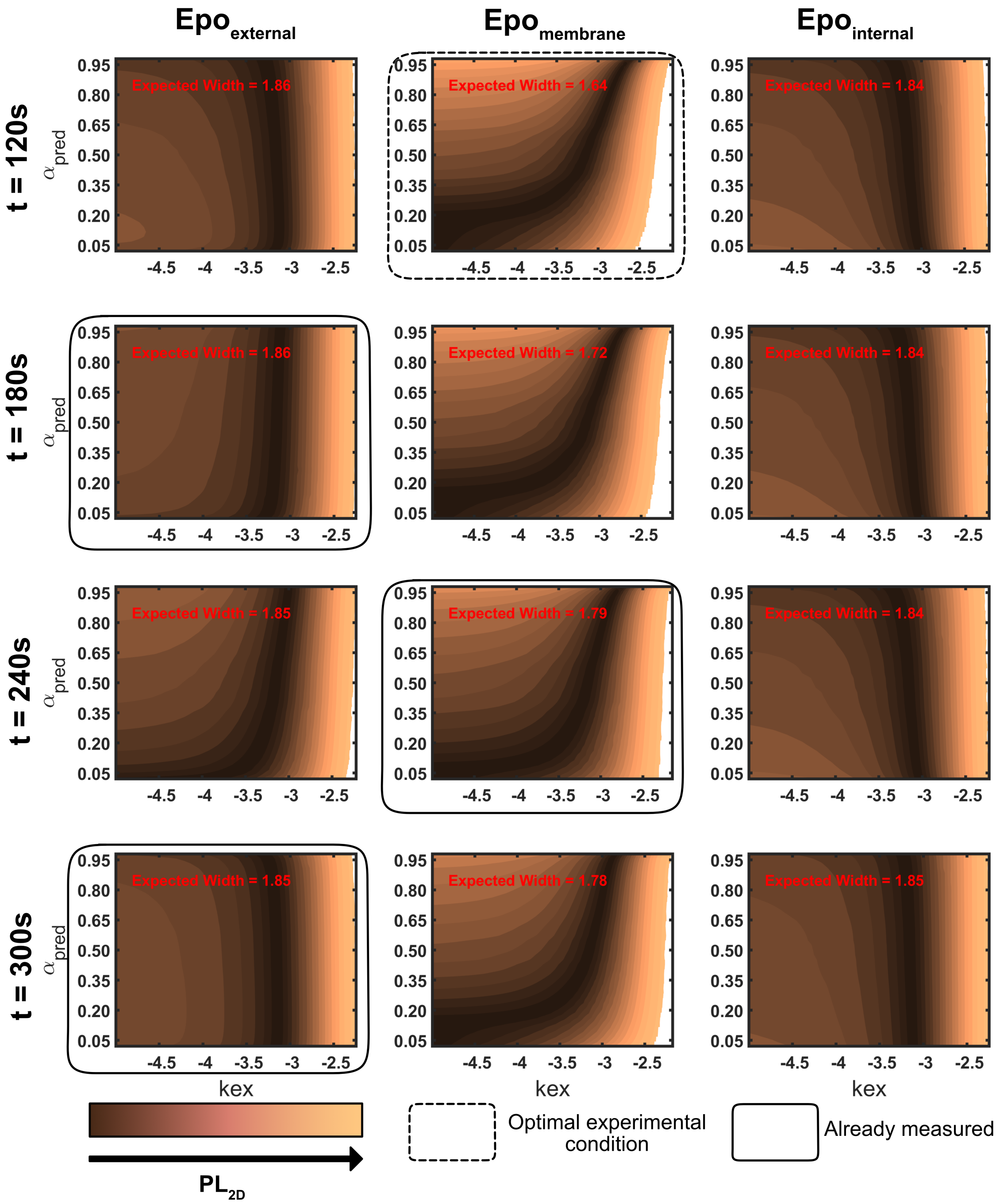

Figure S4. Two-dimensional likelihood profiles for the censored EPO-degradation model for parameter k_{ex}. The different panels correspond to different measurement times (vertical axis) and different observables (horizontal axis). The vertical axis for the individual 2D-profiles indicates prediction confidence levels as in Figure 1D. All measurements will lead to a practically non-identifiable parameter because all possible profile likelihood curves for the parameter hit the bound of the parameter space before crossing the 95%-confidence threshold.
